# Trends in surgery and anesthesia practices during the COVID-19 pandemic: a nationwide analysis from South Korea’s Health Insurance Database

**DOI:** 10.3389/fmed.2025.1548043

**Published:** 2025-06-05

**Authors:** EunJin Ahn, Hyo Jin Kim, Si Ra Bang

**Affiliations:** ^1^Department of Anesthesiology and Pain Medicine, College of Medicine, Chung-Ang University, Seoul, Republic of Korea; ^2^Department of Anesthesiology and Pain Medicine, Chung-Ang University Gwangmyeong Hospital, Gwangmyeong, Gyeonggi, Republic of Korea

**Keywords:** COVID-19, surgery, anesthesia, healthcare, pandemic

## Abstract

**Introduction:**

The coronavirus disease 2019 (COVID-19) pandemic has led to significant changes to global healthcare systems, particularly affecting surgical and anesthetic practices. This study investigated nationwide trend in anesthesia and surgery before and during the pandemic.

**Methods:**

This retrospective, population-based study analyzed confirmed COVID-19 cases in the Korean National Health Information Database from 2019 to 2021. Anesthesia procedures were categorized into general anesthesia, regional anesthesia, and monitored anesthesia care (MAC). COVID-19 positive patients were defined as those with a confirmed diagnosis within 30 days before and after surgery. We analyzed anesthesia modality, patient characteristics, hospital type and COVID-19 trends.

**Results:**

A total of 6,878,556 anesthesia procedures were recorded. The number of procedures decreased slightly in 2020 but increased in 2021. General anesthesia accounted for the majority of procedures. Regional anesthesia, especially brachial plexus block (BPB), and MAC showed an increasing trend. There was a positive correlation between monthly COVID-19 case counts and surgical volume. Most COVID-19 positive patients underwent surgery in general hospitals and received general anesthesia.

**Conclusion:**

Despite the pandemic, overall surgical volume in South Korea remained relatively stable compared to global trends. The shift toward regional techniques like BPB suggests adaptation to reduce aerosol-generating procedures. These findings highlight the need for strategic allocation of resources and preparedness planning in future pandemics.

## 1 Introduction

In late 2019, the global healthcare sector underwent significant transformations due to the coronavirus disease 2019 (COVID-19) pandemic. Healthcare workers encountered numerous challenges, including resource shortages, extended working hours, and psychological strain (1). The focus of healthcare shifted toward controlling the spread of COVID-19, leading to increased healthcare costs and reduced efficiency (2). Moreover, COVID-19 has provided valuable lessons across various aspects of the healthcare field (3). According to an international collaborative research platform for surgeons and anesthesiologists, the initial COVID-19 outbreak precipitated a global economic and social downturn, resulting in the cancelation of millions of surgeries worldwide (4–6). In South Korea, the emergence of COVID-19 in 2020 and 2021 influenced hospital surgery trends and led to changes in the healthcare system (2).

Two previous studies investigated the status of anesthesia services in hospitals across two periods: 2011–2013 and 2014–2016 (7, 8). According to these studies, between 2014 and 2016, general anesthesia was more frequently performed in higher medical institutions, whereas regional anesthesia was induced by non-anesthesiologists at a rate of approximately 11%–16.5%. These studies predate the COVID-19 pandemic and do not capture the changes triggered by this global crisis. Our study aimed to understand the influence of COVID-19 on surgeries and anesthesia practices by analyzing comprehensive data provided by the Korean National Health Insurance System (KNHIS) and the National Health Insurance Sharing Service (NHISS), encompassing a large proportion of operations performed in Korea. These analyses spanned 3 years, ranging from 2019, before the COVID-19 outbreak, to 2021, encompassing the pandemic period.

We examined the changes in the number of surgeries and anesthesia methods used in hospitals to elucidate the impact of COVID-19 on surgical and anesthesia practices. During the peak burden of infectious diseases during the pandemic, there was a significant initial reduction in surgical procedures (5, 6). We aimed to quantify the extent of this decline and determine whether the increase in COVID-19 cases was associated with changes in surgical volume. Through this investigation, we aim to contribute to the appropriate allocation and management of limited medical resources in the event of a pandemic.

The primary objective of this study is to analyze nationwide trends in surgery and anesthesia during the COVID-19 pandemic using the National Health Insurance Database. Specifically, we aim to: (1) quantify the annual and monthly changes in surgical and anesthesia volume from 2019 to 2021; (2) evaluate variations across hospital types and anesthesia modalities; and (3) explore associations between confirmed COVID-19 cases and surgery trends. We hypothesize that regional anesthesia techniques increased in response to infection control concerns, and that surgical care was adapted to accommodate pandemic-related constraints.

## 2 Materials and methods

This study was approved by the Institutional Review Board of Chung-Ang University Gwangmyeong Hospital (No. 2403-149-040). The requirement for informed consent was waived as de-identified administrative claims data were used.

### 2.1 Database

This retrospective, population-based study investigated confirmed COVID-19 cases using the NHID, established by the KNHIS. The NHID contains comprehensive information on healthcare utilization, health screenings, sociodemographic variables, and mortality for the entire population of South Korea. Access to the NHID is granted to researchers with study protocols approved by the official review committee. The KNHIS encompasses claims data from approximately 98% of the Korean population, accounting for nearly 50 million individuals. The NHID includes datasets across various sectors, such as qualification, treatment, medical check-ups, and clinic tables, available as customized or sample research databases. For this study, data were obtained from a customized database of the NHISS that was modified upon request based on our research purpose. It includes information about beneficiaries, such as age, sex, address, and data related to utilized healthcare services, including diagnoses, tests, prescriptions, and procedures (9).

### 2.2 Data extraction and variables

The researchers obtained billing data from 2019 to 2021, encompassing procedure codes for general anesthesia, regional anesthesia, and monitored anesthesia care (MAC). Patients with procedure codes L0101, L1211, L1221, L1212, and L1222 for general anesthesia; L0102, L1213, L1223, L1214, L1224, L1215, L1225, L1216, and L1226 for regional anesthesia; and L0103 and L0104 for MAC were designated as subjects. Patients with COVID-19 were identified by the U071 code (COVID-19 confirmed code) within 30 days before or after surgery.

Study Population and Time Frame We extracted data from January 2019 to December 2021. Eligible patients were those who underwent anesthesia procedures, identified by specific billing codes, at medical institutions during this period. The study period was divided into three phases: pre-pandemic (2019), early pandemic (2020), and extended pandemic (2021).

• Classification of anesthesia

Type Anesthesia procedures were classified into three categories:

1. General anesthesia (e.g., with endotracheal intubation, mask ventilation)
2. Regional anesthesia (e.g., spinal, epidural, brachial plexus block, combined spinal-epidural)
3. Monitored anesthesia care (MAC), including intravenous sedation with spontaneous ventilation

• Exclusion criteria

We excluded cases with overlapping or multiple anesthesia codes (> 2 types per case), and those with both upper and lower body regional techniques in a single session. Local infiltration anesthesia was not included.

• Hospital classification

Hospitals were classified according to South Korea’s medical law. The study also examined the number of hospitals with anesthesiologists according to institution type.

○ Clinics: < 30 beds
○ Hospitals: 30–100 beds
○ General hospitals: > 100 beds
○ Tertiary referral hospitals designated by the government as specialized hospitals, which perform educational functions as tertiary referral hospitals.

We collected information on anesthesia methods (including general anesthesia, regional anesthesia, upper limb nerve block anesthesia, and MAC), American Society of Anesthesiologists (ASA) Physical Status > 3 (ASA3 +), emergency surgery status, COVID-19 confirmation date, sex, age, region, hospital type, and the presence of anesthesia consultants from the patients. The yearly and monthly frequencies of these variables were analyzed.

Coronavirus disease 2019 confirmation was defined by the ICD-10 code U071 recorded within 30 days before or after surgery (10). This 30 days window was chosen to account for both the incubation period of the virus and potential delays in post-operative diagnosis. Our definition is supported by CDC findings, which indicate that most individuals recover from acute COVID-19 within 4 weeks, thereby allowing us to capture both the immediate and short-term effects of the infection on surgical and anesthetic outcomes (11). The number of confirmed COVID-19 cases by year and month was obtained from the World Health Organization’s COVID-19 Explorer website^1^.

### 2.3 Statistical analysis

Descriptive statistics were used to report annual and monthly trends. Correlations between COVID-19 case numbers and surgery volume were evaluated using scatter plots and correlation coefficients. Data were analyzed Statistical analyses were conducted using R software (version 4.2.2) and SAS Enterprise Guide (version 6.1; SAS Institute Inc., Cary, NC, United States).

## 3 Results

**FIGURE 1:**
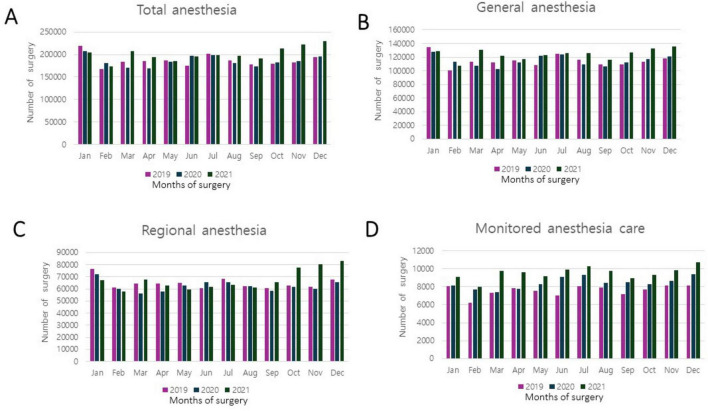
Number of surgeries performed with anesthesia during the coronavirus disease 2019 (COVID-19) pandemic: monthly and yearly variations. **(A)** Total number of cases, including general anesthesia, regional anesthesia, and monitored anesthesia care. **(B)** Number of general anesthesia cases. **(C)** Number of regional anesthesia cases. **(D)** Number of monitored anesthesia care cases.

We examined the changes in anesthesia types from 2019 to 2021, categorized by year and month (Figure 1). Upon comparing the monthly data, the total number of anesthesia procedures in 2019 was highest in January and July; contrastingly, in 2021, it peaked in November and December. Over the 3 years, the fewest surgeries were performed in February. The monthly trends for general anesthesia closely mirrored the overall change in the total number of anesthesia procedures. The number of regional anesthesia procedures increased during the winter months, whereas that of MAC procedures was slightly higher in summer and winter than in spring and autumn.

**FIGURE 2:**
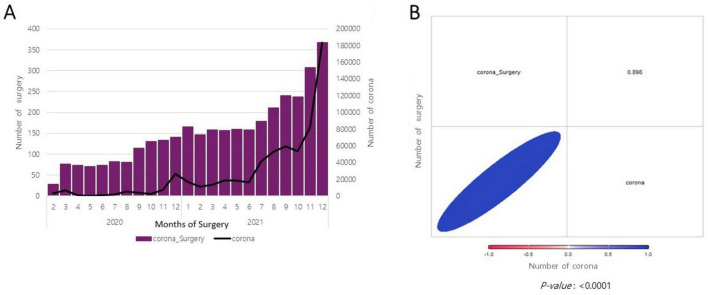
Correlation between the number of surgeries performed among coronavirus disease 2019 (COVID-19)-confirmed cases and the number of patients with COVID-19. **(A)** Monthly changes in the number of surgeries performed among COVID-19-confirmed cases and the number of patients with COVID-19. **(B)** Scatter plot.

The changes in the number of patients with COVID-19 who underwent surgery were analyzed (Figure 2). The correlation between the number of confirmed COVID-19 cases and the number of surgeries for patients with COVID-19 was examined, with a positive correlation observed between the number of patients with COVID-19 and the number of surgeries performed in 2020 and 2021.

**FIGURE 3:**
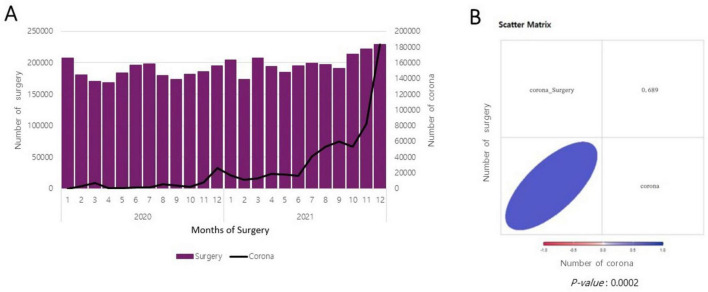
Correlation between the number of surgeries performed and the number of patients with coronavirus disease 2019 (COVID-19). **(A)** Monthly changes in the number of surgeries performed during the COVID-19 era and the number of patients with COVID-19. **(B)** Scatter plot.

The correlation between the number of confirmed COVID-19 cases and the total number of surgeries performed is illustrated in Figure 3. A positive correlation was observed between the number of confirmed COVID-19 cases and the total number of surgeries performed in 2020 and 2021.

The total number of anesthesia procedures decreased slightly in 2020 but increased in 2021. Monitored anesthesia care (MAC), intravenous general anesthesia, mask ventilation, and BPB anesthesia showed a steady upward trend in number, whereas anesthesia requiring endotracheal intubation and spinal anesthesia decreased in 2020 but increased again in 2021. Epidural anesthesia was induced less often in 2020, but only slightly less often in 2021, than in 2019. We analyzed the number of patients with COVID-19 who were administered anesthesia during the COVID-19 pandemic. In 2020, when the pandemic began to spread significantly in South Korea, 1,008 patients with COVID-19 were administered anesthesia, with the majority undergoing general anesthesia requiring endotracheal intubation. Among them, 10.7% underwent spinal anesthesia and 2.6% underwent BPB. In 2021, 2,490 patients with COVID-19 were induced anesthesia: 72.9% underwent general anesthesia with endotracheal intubation, 17.1% underwent spinal anesthesia, and 3.8% underwent BPB (Table 1).

**TABLE 1:** Annual changes in the type of anesthesia induced in all patients and in patients with COVID-19.

Type of anesthesia	2019	2020	2021	Total
MAC	91,256	101,028	114,581	306,865
COVID-19	–	13 (1.3)	16 (1.6)	–
General	1,374,646	1,374,825	1,492,103	4,241,574
COVID-19	–	857	1,864	–
IV-General	163,787	167,554	178,130	509,562
COVID-19	–	2 (0.2)	7 (0.7)	–
Endotracheal	1,059,894	1,049,418	1,142,038	3,251,350
COVID-19	–	817 (81.1)	1,817 (72.9)	–
Mask	150,874	157,853	171,935	480,662
COVID-19	–	38 (3.8)	40 (1.6)	–
Regional	775,129	747,869	807,119	2,330,117
COVID-19	–	138	610	–
IV-Regional	18,665	20,101	19,679	58,445
COVID-19	–	6 (0.6)	1 (0.1)	–
SA	434,912	413,460	462,047	1,310,419
COVID-19	–	103 (10.7)	427 (17.1)	–
EA	121,433	108,549	113,918	343,900
COVID-19	–	1 (0.1)	85 (3.4)	–
BPB	189,559	197,310	203,324	590,193
COVID-19	–	26 (2.6)	94 (3.8)	–
CSE	10,560	8,449	8,151	27,160
COVID-19	–	2 (0.2)	3 (0.1)	–
Total	2,241,031	2,223,722	2,413,803	6,878,556
COVID-19	–	1,008	2,490	–

Data are presented as the number of patients (and percentage, where applicable). COVID-19, coronavirus disease 2019; MAC, monitored anesthesia care; Mask, general anesthesia performed with mask ventilation; SA, spinal anesthesia; EA, epidural anesthesia; BPB, brachial plexus block; CSE, combined spinal–epidural anesthesia.

We compared the number of anesthesia procedures over 3 years, categorized by hospital type (Table 2). Tertiary referral and general hospitals experienced a temporary decline in 2020, followed by an increase in 2021. However, general hospitals and clinics experienced a steady increase in anesthesia procedures. In 2020, the number of anesthesia procedures performed on patients with COVID-19 was as follows: tertiary referral hospitals induced anesthesia in 310 out of 1,008 patients, accounting for 30.8% of cases; general hospitals handled 67.9% of cases, and hospitals were responsible for 1.3% of cases. In 2021, the percentage of COVID-19 anesthesia cases attended to at tertiary referral hospitals decreased to 20.9%, whereas general hospitals accounted for 74.9%, and hospitals were responsible for 4.2% (Table 2).

**TABLE 2:** Annual changes in the number of anesthesia procedures conducted in all patients and in patients with COVID-19 according to the type of hospital.

Class of institution	2019	2020	2021
Tertiary referral hospitals	582,657	554,962	597,073
COVID-19	–	310 (30.8)	520 (20.9)
General hospitals	669,838	675,421	728,392
COVID-19	–	685 (67.9)	1,866 (74.9)
Hospitals	740,430	731,228	772,280
COVID-19	–	13 (1.3)	104 (4.2)
Clinics	248,106	262,111	316,058
COVID-19	–	0	0
Total	2,241,031	2,223,722	2,413,803
COVID-19	–	1,008	2,490

Data are presented as the number of patients (and percentage, where applicable). Hospitals with fewer than 30 beds were categorized as clinics, those with 30–100 beds as hospitals, those with more than 100 beds as general hospitals, and those with more than 100 beds designated by the government as specialized hospitals, which perform educational functions as tertiary referral hospitals. COVID-19, coronavirus disease 2019.

We analyzed variations in anesthetic techniques across different hospital types (Table 3). Initially, there was a decline in general anesthesia cases, followed by a resurgence in tertiary referral hospitals. Conversely, general hospitals and clinics exhibited a consistent uptrend in general anesthesia cases, whereas the count of regional anesthesia cases dropped in 2020, only to rise thereafter.

**TABLE 3:** Changes in anesthetic technique according to the type of hospital.

Class of institution	2019			2020			2021		
	GA	RA	MAC	GA	RA	MAC	GA	RA	MAC
Tertiary referral hospital	519,294 (37.8)	42,739 (5.5)	20,624 (22.6)	494,052 (35.9)	39,570 (5.3)	21,340 (21.1)	532,321 (35.7)	42,260 (5.2)	22,492 (19.6)
COVID-19	–	–	–	274	26	10	443	67	10
General hospital	429,140 (31.2)	215,099 (27.7)	25,599 (28.0)	441,752 (32.1)	203,784 (27.2)	29,885 (29.6)	487,702 (32.7)	208,187 (25.8)	32,503 (28.4)
COVID-19	–	–	–	580	103	2	1,396	464	6
Hospital	285,707 (20.8)	423,392 (54.6)	31,331 (34.3)	286,836 (20.9)	412,487 (55.1)	31,905 (31.6)	304,217 (20.4)	432,148 (53.5)	35,915 (31.3)
COVID-19	–	–	–	3	9	1	25	79	0
Clinic	140,505 (10.2)	93,899 (12.1)	13,702 (15.0)	152,185 (11.1)	92,028 (12.3)	17,898 (17.7)	167,863 (11.3)	124,524 (15.4)	23,671 (20.6)
COVID-19	–	–	–	0	0	0	0	0	0
Total	1,374,646	775,129	91,256	1,374,825	747,869	101,028	1,492,103	807,119	114,581
COVID-19	–	–	–	857	138	13	1,864	610	16

Data are presented as the number of patients (and percentage, where applicable). Hospitals with fewer than 30 beds were categorized as clinics, those with 30–100 beds as hospitals, those with more than 100 beds as general hospitals, and those with more than 100 beds designated by the government as specialized hospitals, which perform educational functions as tertiary referral hospitals. COVID-19, coronavirus disease 2019. GA, general anesthesia; RA, regional anesthesia; MAC, monitored anesthesia care.

In 2020, among ASA3+ patients, 0.2% tested positive for COVID-19, with similar percentages observed among patients receiving emergency and nighttime surgeries (0.2% and 0.3%, respectively). By 2021, there was a progressive rise in COVID-19 cases among ASA3+ patients, reaching 0.4%. Although the percentage among patients receiving emergency surgeries remained constant at 0.3%, it increased to 0.9% among patients receiving nighttime surgeries.

## 4 Discussion

In this study, the overall number of surgeries declined slightly from 2019 to 2020 and subsequently increased in 2021. Notably, Japan experienced a significant decline in general anesthesia cases around May 2020 (12), whereas South Korea witnessed a reduction in March and April 2020, followed by a resurgence after May. This overall fluctuation was largely influenced by a decline and subsequent increase in regional anesthesia procedures. The frequency of cancer procedures, typically deemed high priority, declined as patients opted for alternative treatments, such as targeted therapies, radiation, and neoadjuvant chemotherapy. Additionally, procedures for lower-risk cancers, such as prostate cancer or stage 0 breast cancer, were postponed from 15 March to 2 May 2020 (5).

Among the annual anesthetic data totaling 2,129,871 cases in 2013, general anesthesia accounted for 55%, regional anesthesia for 36%, and MAC for 9%. Most general anesthesia procedures (80%) were performed in general hospitals, whereas regional anesthesia procedures (60%) were more prevalent in hospitals or clinics with fewer than 100 beds (8). From 2014 to 2016, approximately 2 million anesthesia procedures were performed: general anesthesia (53.4%), regional anesthesia (37.8%), and MAC (8.8%) (7). The present study, covering 2019–2021, reflects minimal deviation from previous data but incorporates changes attributed to the COVID-19 pandemic, particularly a noticeable increase in regional techniques such as BPB and MAC. This shift suggests a deliberate move to minimize aerosol-generating procedures for COVID-19–positive patients to reduce viral transmission. Our data showed a steady increase in BPB and MAC procedures across all hospital types, with particularly notable uptake in general hospitals and clinics. This trend may reflect heightened awareness of infection control strategies and clinical adaptations to reduce airway manipulation, especially in settings with limited airborne precaution resources.

In this study, considering the incubation period of COVID-19, [typically 1–14 days (10)], patients who tested positive within 30 days post-surgery were defined as patients with COVID-19. The number of patients who developed COVID-19 after surgery more than doubled in 2021 compared to 2020 (2,490 vs. 1,008). Most patients with COVID-19 (67.9% in 2020 and 74.9% in 2021) underwent surgery in general hospitals with over 100 beds, and most were administered general anesthesia (580 out of 685 in 2020 and 1,396 out of 1,866 in 2021). The proportion of patients with COVID-19 who were administered general anesthesia also increased from 0.2% to 0.4% among severe cases, and the number of patients undergoing nighttime surgeries increased more than threefold. As the spread of COVID-19 escalated, the number of patients with COVID-19 undergoing surgery increased. This may have been a logical consequence of the widespread impact of the pandemic.

The declining proportion of COVID-19 surgical cases in tertiary hospitals, accompanied by a concurrent increase in general hospitals, suggests a redistribution of care—potentially reflecting deliberate decentralization policies or capacity limitations in tertiary centers. This shift is further supported by trends in surgical volume, as tertiary referral hospitals initially saw a decrease before gradually recovering. Notably, a similar pattern was observed across different types of anesthesia, with tertiary hospitals showing a decline in both the number and proportion of surgeries performed under general anesthesia, regional anesthesia, and MAC compared to other hospitals in 2020 and 2021.

The increase in ASA3+ patients, along with a rise in nighttime and emergency surgeries among COVID-19–positive patients, indicates a shift toward higher-acuity surgical care during the pandemic. This trend may reflect delayed patient presentations, reduced access to elective procedures, or reprioritization of hospital resources. Notably, the proportion of ASA3+ patients more than tripled in 2020 and 2021 compared to 2019, accompanied by a marked increase in urgent and nighttime surgeries. These findings suggest a growing burden of severe cases in tertiary referral hospitals, potentially increasing the risk for both patients and medical staff, and may explain the overall decline in surgical volume or proportion at these institutions (13, 14). Taken together, our results underscore the importance of a flexible and responsive anesthesia workforce to adapt to changing clinical demands during a healthcare crisis.

Unexpected positive correlation with COVID-19 cases one notable finding was the positive correlation between COVID-19 case numbers and surgical volume. This initially counterintuitive result may be explained by several factors: an increase in urgent and emergent procedures; improved triage and infection control allowing continued surgeries; or increased system capacity over time. Further investigation into case types and severity would be needed to confirm these hypotheses.

Numerous studies have documented a significant increase in canceled surgeries and the adoption of alternative treatments with the spread of COVID-19 (1, 5, 6, 12). According to Ghoshal’s et al. (15) study, the number of surgical procedures was significantly reduced during the peak of the pandemic and did not fully recover to pre-pandemic levels until 2021 (14). A study published in JAMA reported that in the United States, particularly in April 2020, the surgical volume decreased by up to 48% compared to that in 2019. Notably, surgeries related to ear–nose–throat and musculoskeletal issues declined but steadily increased to pre-pandemic levels after July 2020 (5). In contrast, data from South Korea showed a less pronounced decline in the total number of surgeries performed in April 2020 compared to 2019. The decrease was relatively modest, with recovery starting as early as May and June, even demonstrating an increase in surgery volume compared with the previous year. Although this trend could partially be attributed to an overall increase in surgery volume over time, as suggested by previous studies (7, 8), the spread of COVID-19 did not result in a decrease in overall surgeries. This suggests that, despite the challenges posed by the pandemic, other factors may have contributed to the observed increase in surgeries.

The spread of COVID-19 has impacted surgical practices and anesthesia techniques. Both anesthesiologists and surgeons are at risk of exposure to the patient’s virus, prompting extensive discussions on strategies to mitigate this risk (16). It has been highlighted that the virus present in the pneumoperitoneum may be released during surgery, potentially contaminating both the surgical team and the operating environment. In the absence of adequate precautions to manage this risk, open surgery should be considered as a safer alternative (17). To minimize the risk of transmission, it tis recommended to lower the power setting of electrocautery, use small port incisions, maintain low CO_2_ pressure during the laparoscopic surgery, and ensure close evacuation of all gas at the end of the procedure (18). In some guidelines, it has been recommended to delay cancer surgery for up to 3 months, depending on the biology of the cancer (19).

Several guidelines have been issued concerning anesthesia methods (12, 20–22). In the initial phases of the pandemic, infected patients were deemed an inevitable demographic for anesthesiologists. In some studies, general anesthesia was advocated, except for cesarean delivery (12, 23). Antonio’s research has demonstrated that neuraxial anesthesia for managing parturients with SARS-CoV-2 infection is safe for both patients and healthcare workers (24, 25). This recommendation was based on the lower risk of healthcare worker exposure during surgeries performed under general anesthesia, as opposed to regional or local anesthesia, where aerosols from infected patients could potentially be spread through their respiratory tracts. Moreover, minimizing bag–mask ventilation via rapid sequence induction can mitigate the risk of aerosolizing airway secretions (12). Following surgery, bypassing the post-anesthesia care unit and transferring the patient to an isolated room for extubation was recommended to minimize exposure to patient secretions during this process. In contrast to the previous viewpoint, aerosol-generating procedures, such as endotracheal intubation and coughing during extubation, pose a significant risk of infection to healthcare workers, with infection rates increasing more than 6-fold (26). Moreover, regional anesthesia may emerge as the preferred option for patients with COVID-19 owing to its association with improved outcomes (19, 20). However, COVID-19 frequently correlates with thrombocytopenia; thus, platelet counts should be evaluated before inducing regional anesthesia. In cases of low platelet counts, a nerve block may be preferable (12).

This study revealed an increase in the induction of regional anesthesia in 2020 and 2021. The induction of spinal anesthesia decreased in 2020 but rebounded in 2021, whereas the use of nerve blocks, such as BPB, steadily increased in both years. This trend suggests a preference for nerve blocks, possibly due to recommendations favoring their use. However, nerve blocks require ultrasound guidance and skilled practitioners, making their adoption dependent on the available medical resources and expertise. The ERAS (Enhanced Recovery After Surgery) protocol is an approach that includes various management strategies before, during, and after surgery to promote patient recovery (27). This protocol addresses key elements such as pain management, nutrition management, early mobilization, and prevention of postoperative complications. As the ERAS protocol gains attention in healthcare institutions, the use of regional anesthesia is expected to increase gradually (28).

This study had some limitations. First, the use of administrative claims data from the National Health Insurance Database may lack detailed clinical information such as comorbidities, exact surgical indications, and perioperative outcomes. As a result, we were unable to perform risk-adjusted or case-mix analyses, nor stratify surgeries by urgency (e.g., oncologic vs. elective vs. trauma). Second, the identification of COVID-19–positive patients was based on diagnosis codes within 30 days before or after surgery. This broad window, although informed by existing literature on incubation and detection timing, may have led to misclassification in certain cases, especially during the early phases of the pandemic when testing strategies were still evolving. Although this serves as a limitation by not including patients’ clinical information, it also offers the strength of including a large volume of data and confirming prescriptions. Additionally, changes in specific departments could not be assessed, which should be considered in future studies.

Coronavirus disease 2019, with its highly infectious nature and prolonged incubation period, presents a constant challenge in surgical settings, as healthcare professionals always must face the possibility of encountering patients with COVID-19. In cases requiring endotracheal intubation for general anesthesia induction, the risk of COVID-19 transmission is significantly high, requiring healthcare providers to be prepared. It is difficult to gauge the exact extent of the patient’s condition and the associated medical sacrifice. However, patients with COVID-19 are an unavoidable group for anesthesiologists. COVID-19 has undoubtedly been the most impactful viral disease on healthcare in the 21st century. However, there is no guarantee that similar pandemic viral diseases will not emerge. Therefore, implementation of national or international standards of intraoperative care in each country will be need such as “the Top 10 Anesthesia Patient Safety Issues Worldwide” (29). Also, we must build better systems based on past experiences to advance healthcare.

## Data Availability

The datasets presented in this article are not readily available because data are not publicly available due to institutional policy. Further information on how to request these data can be found at the National Health Insurance Sharing Service website (https://nhiss.nhis.or.kr/bd/ab/bdaba000eng.do). Requests to access the datasets should be directed to the National Health Insurance Sharing Service website (https://nhiss.nhis.or.kr/bd/ab/bdaba000eng.do).

## Footnotes

1
https://worldhealthorg.shinyapps.io/covid/
